# Diving into the hidden viral world of marine protists

**DOI:** 10.1128/jvi.01262-25

**Published:** 2026-01-05

**Authors:** Kayla Surgenor, Craig McCormick

**Affiliations:** 1 Department of Microbiology & Immunology, Dalhousie University 3688, Halifax, Nova Scotia, Canada; Indiana University Bloomington, Bloomington, Indiana, USA

**Keywords:** mirusvirus, protist, metagenomics, persistence, carbon cycle, thraustochytrid, polyunsaturated fatty acids

## Abstract

As the most abundant biological entities in the ocean, viruses of microbes play important roles in regulating host population dynamics and influencing biogeochemical cycles. Metagenomic surveys have revealed an astounding reservoir of viral genetic diversity in single-celled marine eukaryotes known as protists, but the vast majority of these viruses have not been directly observed, and information about their protist hosts remains fragmentary. The 2023 discovery of mirusviruses provides a striking example, whereby metagenomic surveys of samples collected by the *Tara* Oceans expedition led to the discovery of a new phylum of viruses, the *Mirusviricota*, with remarkable chimeric genomes encoding structural proteins from herpesviruses and enzymes from giant eukaryotic viruses. However, because mirusviruses were detected indirectly by metagenomics, their host range remained unclear, and their biological properties unexplored. Here, we provide new insights into research approaches to identify *bona fide* protist hosts for marine viruses and characterize virus-host interactions. A greater understanding of these viruses and their natural hosts will unlock opportunities to understand the roles that they play in regulating biogeochemical processes in marine habitats.

## AN EXPLOSION OF NEW VIRUS DISCOVERIES IN MARINE ENVIRONMENTS

Oceans harbor immense biological diversity, but only a fraction of marine life has been described. As on land, viruses of microbes are major players in marine ecosystems where they regulate host population diversity and community structure through predation (1). Beyond predator-prey relationships, marine viruses also influence carbon cycling in the ocean by controlling the biological carbon pump, releasing organic matter by killing phytoplankton and heterotrophic bacteria, and sequestering it in the deep ocean via sinking cell aggregates (2, 3). Despite evidence for substantial impact on biogeochemical cycles, our understanding of these systems remains limited due to a lack of fundamental knowledge about interactions between marine viruses and their hosts.

**Fig 1:**
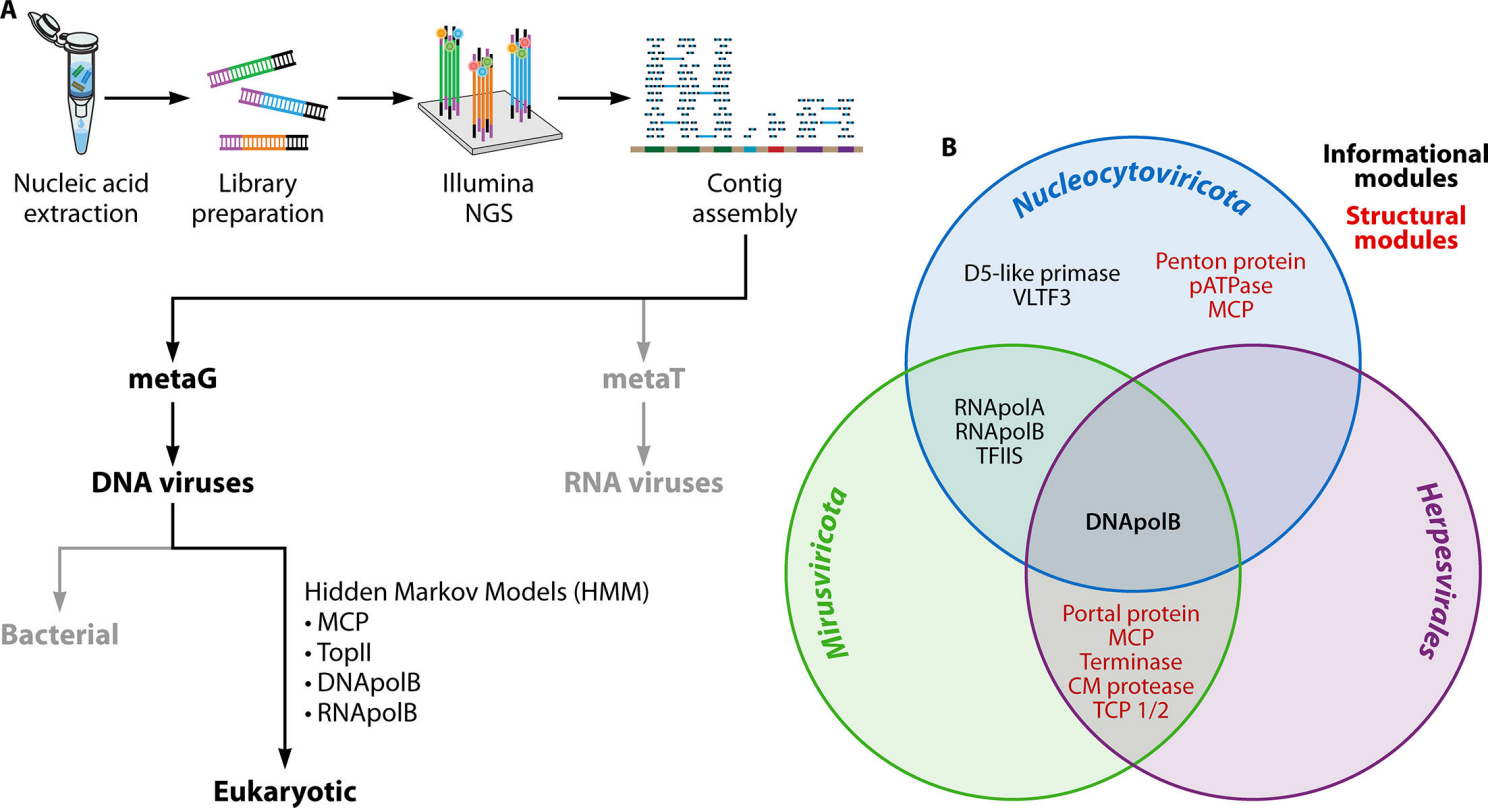
The hunt for marine viruses. (**A**) Flowchart of nucleic acid database curation and analysis used to identify and characterize marine viruses from metaG or metaT data sets. (**B**) Classification of core *Mirusviricota* genes. *Mirusviricota* is a chimera of structural and information modules from *Herpesvirales* and *Nucleocytoviricota*. Adapted from Gaia et al. (12). Abbreviations: capsid maturation (CM) protease; family B DNA polymerase (DNApolB); major capsid protein (MCP); DNA-dependent RNA polymerase subunits A (RNApolA) and B (RNApolB); triplex capsid protein (TCP); transcription factor II-S (TFIIS); topoisomerase family II (TopII); viral late transcription factor 3 (VLTF3).

The revolution in next-generation sequencing (NGS) technologies that dramatically increased processivity and lowered costs enabled analysis of marine microbial communities in unprecedented detail through metagenomics. This is exemplified by the *Tara* Oceans project, an interdisciplinary expedition that set sail in 2009 to characterize the diversity and interactions of marine plankton, including single-cell eukaryotes known as protists (4). Over its 3-year voyage, *Tara*’s team sampled various depths at over 200 locations across the world’s oceans. Each sample was filtered to separate microbes by size, then preserved for further analysis on land, where nucleic acids were extracted for library preparation and sequenced with Illumina technology for metabarcoding (metaB), metatranscriptomic (metaT) or metagenomic (metaG) analysis (5) (Fig. 1A). MetaG data sets from *Tara* Oceans have been analyzed extensively to provide insight into viruses found in diverse marine habitats worldwide. Assembly of viral metagenomes from virus-enriched (<0.22 µm) fractions identified ~200,000 viral metagenomes, illustrating the transformational impact of large metagenomic studies (6–8). These initial studies only analyzed viruses smaller than 0.22 μm and were heavily biased towards double-stranded (ds) DNA phages. By contrast, metaT approaches have been instrumental in the discovery of RNA viruses that largely infect bacteria in marine environments (9–11). These foundational discoveries provided extraordinary momentum, supporting the discovery and characterization of additional marine viruses.

Advances in metagenomics also led to an explosion of eukaryotic virus discovery, many of which are members of the large virus phylum, *Nucleocytoviricota*. Pioneering metagenomic studies of deep-sea vents led to the discovery of many giant viruses, expanding the number of eukaryotic viral genomes (13). Large-scale metagenomic approaches from the *Tara* Oceans expeditions built on these foundational studies by searching for giant virus sequences from eukaryotic fractions. Following contig assembly, Hidden Markov Models (HMMs) were used to screen for shared viral reference genes, such as major capsid proteins (MCPs), to generate eukaryotic virus metagenomic-assembled genomes (MAGs) (14, 15). These MAGs were then combined with additional data to create the Global Ocean Eukaryotic Viral (GOEV) database (12), revealing the extraordinary diversity of viruses that infect marine eukaryotes.

The viruses of the phylum *Nucleocytoviricota*, which have gained great attention and are commonly known as “giant viruses,” are abundant in marine environments where they infect diverse hosts, including protists (12, 16, 17). Analysis of data from the *Tara* Oceans project led to the discovery of an additional giant virus phylum, the *Mirusviricota*, which features information module genes related to those of the *Nucleocytoviricota* and structural genes related to those from the *Duplodnaviria* (12). Thus, the *Mirusviricota* are true chimeras, with giant virus enzymes that direct the replication of a dsDNA genome and packaging into herpesvirus-like capsids (Fig. 1B). These viruses are currently classified as members of the *Duplodnaviria* due to MCP similarity. The *Mirusviricota* represent the first known members of *Duplodnaviria* to infect single-cell eukaryotes, expanding the host range of *Duplodnaviria*. Additionally, metaG and metaT analyses revealed that mirusviruses are both abundant and active in the epipelagic zone. The discovery of mirusviruses provides a unique link between *Nucleocytoviricota* and *Duplodnaviria* and is yet another transformational impact of the *Tara* Oceans project.

Further work to identify and classify viruses from the *Tara* Oceans metagenomic data set revealed community dynamics of marine viruses. Analysis of dsDNA reference genes, such as Topoisomerase family II (*TopII*) and family B DNA polymerase (*polB*), revealed that *Nucleocytoviricota* species are abundant in every ocean on Earth, particularly in the epipelagic zone (18, 19). Some viruses are ubiquitous in the oceans, with enrichment of distinct viral communities in certain ecological zones (19). The most unique viral communities can be found in harsh environments, such as the Arctic Ocean, generally matching the distribution of distinct host species that inhabit this region (20, 21). Viruses are obligate parasites; therefore, their distribution is dependent on the distribution of their hosts (19, 22) but can also be affected by abiotic factors, such as temperature, nutrients, and ultraviolet radiation (23, 24), as well as seasonality (25).

The *Tara* Oceans project led to the identification of many novel marine viruses with diverse properties. While some newly discovered viruses have conventional lytic replication cycles, there have also been reports of integrated and transcriptionally active endogenous viral elements (EVEs) in marine protist genomes, suggesting a latent infection strategy (26, 27). Unlike typical EVEs with partially or extensively degraded genomes that reflect ancient integration events and fixation (28), these EVEs can support the production of viral particles. However, the regulation of EVE gene expression and dynamics of virus production and spread remains largely unclear in these marine protist hosts.

Marine viruses infect various hosts, such as photosynthetic and heterotrophic eukaryotes. Traditional methods to identify hosts for marine viruses have relied upon culture of candidate hosts in the laboratory and identification of viral gene products and viral particles. The advent of metagenomic sequencing approaches has accelerated the rate of discovery of marine viruses as well as the identification of candidate hosts via co-occurrence (29). This discovery method is nicely complemented by single-cell RNA sequencing (scRNAseq), whereby single candidate host cells are isolated, and cellular and viral RNA is extracted, reverse-transcribed, and sequenced (30). This method can help identify infected cells from mixed populations of cells in marine environmental samples or laboratory cultures (31). After *bona fide* hosts are identified, scRNAseq can provide rich information about viral gene expression and host responses in heterogeneous host cell populations (31, 32), which is particularly valuable for protist hosts with complex life cycles.

## THE IMPACT OF VIRUSES ON PROTIST METABOLISM

A hallmark of virus infection is the manipulation of the host cell to create an intracellular environment favorable to viral replication, a concept known as the “virocell” (33). To construct their virocell, giant marine viruses express a repertoire of auxiliary metabolic genes (AMGs) that reprogram the host cell metabolic landscape to support virus replication. Many viruses have extensive repertoires of AMGs that exert control over nodal cellular processes. Putative viral AMGs with homology to known enzymes that control amino acid metabolism, carbon metabolism, and nucleic acid processing have been identified (15). Many unique AMGs are found in Arctic and deep ocean viruses, allowing them to adapt to these unique environmental conditions (20, 34, 35). The repertoire of giant virus AMGs is extensive, many of which are beyond the scope of this review but are covered extensively by Moniruzzaman et al. (36). Numerous viral AMGs were acquired through horizontal gene transfer events during long co-evolutionary history with diverse hosts (37).

A feature of the virocell is increased energy production to meet the demands of viral replication. These cells feature dysregulation of carbon metabolism, bypassing rate-limiting steps in glycolysis, tricarboxylic acid (TCA), and beta-oxidation pathways, favoring viral replication (15, 38). Virus infection may also increase the uptake efficiency of nutrients, such as nitrogen, phosphorus, and sulfur, from the environment through the expression of viral nutrient transporters (15). Increasing the uptake of scarce nutrients provides a competitive advantage over uninfected cells while also creating a favorable environment for virus replication. Giant viruses also encode accessory genes to fine-tune the virocell, influencing other processes not directly related to metabolism. For example, the lytic Emiliania huxleyi viruses (EhVs) that control blooms of *Gephyrocapsa huxleyi* (formerly *Emiliania huxleyi*) in marine habitats encode a near-complete sphingolipid synthesis pathway that may be involved in remodeling host cell membranes (39, 40). This dramatic refashioning of lipid metabolism provides a convenient way to identify infected hosts. Importantly, the viral processes that direct virocell formation are dynamic, with the ability to adapt to different environmental conditions, suggesting complex regulatory mechanisms (41).

Giant viruses encode accessory genes that regulate cell fate. Chloroviruses encode copper-zinc superoxide dismutases that convert cytotoxic reactive oxygen species (ROS) into hydrogen peroxide, promoting the survival of infected cells and extending windows of productive viral replication (42). By contrast, EhV encodes a remarkable collection of enzymes that direct the production of cytotoxic glycosphingolipids that trigger programmed cell death to aid release of viral progeny (43). By triggering cell death, these viral proteins influence carbon availability on a global scale, depleting it from higher trophic levels and causing it to move down the water column, where it supports aphotic ecosystems (44). This is a central mechanism in viral regulation of biogeochemical cycling in the oceans.

## HOST DEFENSES AND VIRAL COUNTERMEASURES

Virus infection occurs throughout all domains of life, thereby influencing the evolution of all living organisms (45, 46). Consequently, all life has also evolved defense mechanisms to counteract viral infection, although antiviral strategies vary dramatically (47). Studies on the evolution and diversity of antiviral defenses have gained significant momentum, fueled by discoveries, such as the prokaryotic CRISPR-Cas phage defense system (48–50), and conservation of nucleotide-editing Viperin across domains of life, including in the green algae group Chlorophyta (51). Protist antiviral defenses have primarily been examined through an evolutionary immunology perspective using choanoflagellates, single-cell eukaryotes that share a specific common ancestry with animals. Choanoflagellate protists share several antiviral defense systems with animals, centered on the detection of foreign nucleic acids, such as Toll-like receptors (TLRs) (52) and cGAS-STING pathways (53). However, the diversity of protist antiviral defenses beyond choanoflagellates is not well understood due to paraphyletic groupings that lack a common ancestor. Additionally, the role of antiviral systems against the cognate viruses of protists is unclear. The recent dramatic increase in available genomes of marine protists and their viruses, facilitated in part by the *Tara* Oceans project, provides unique opportunities when paired with laboratory models to examine the complexity and evolution of antiviral defense systems in single-cell eukaryotes.

**Fig 2:**
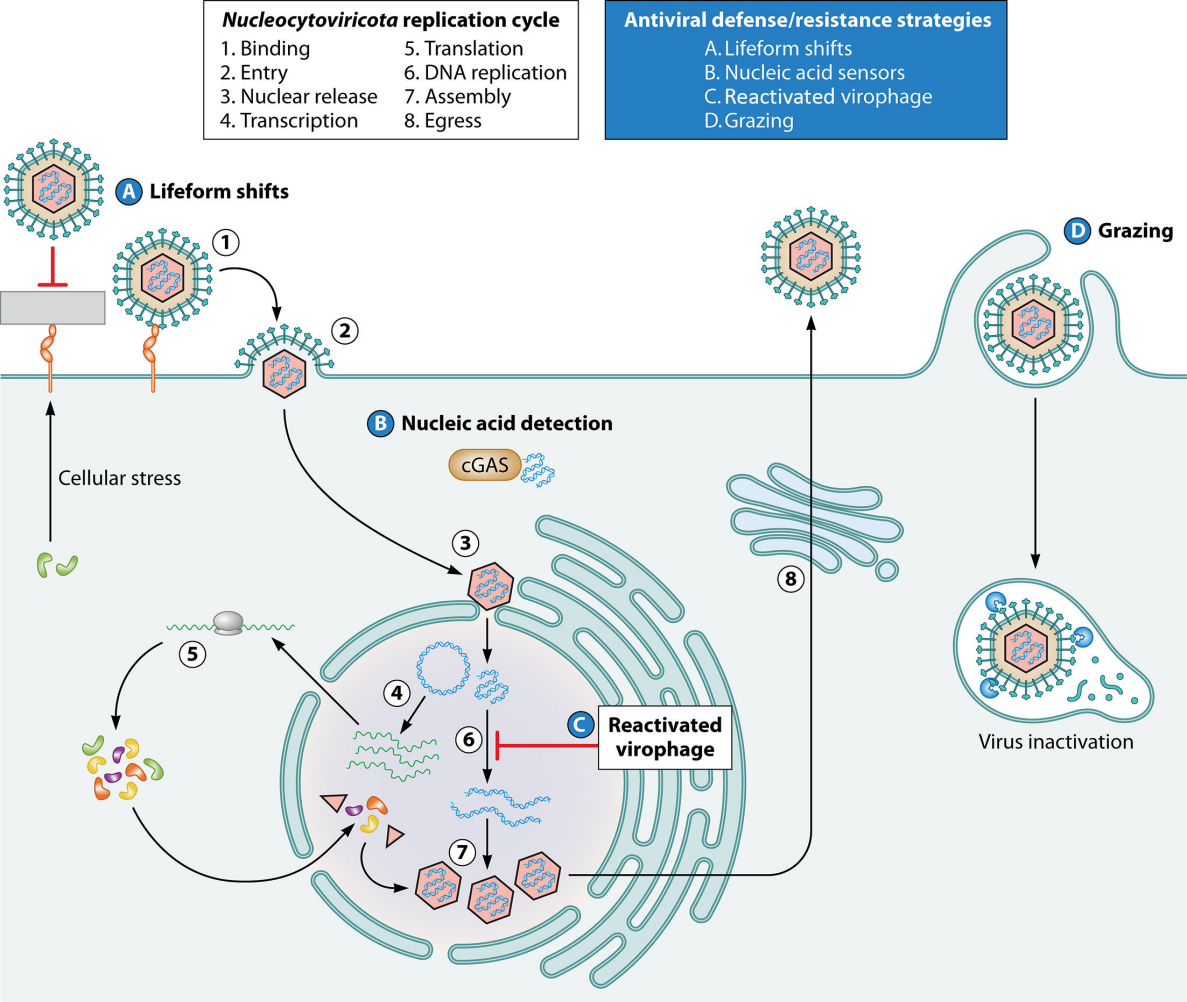
Host defense and resistance strategies against viral infection. Generalized *Nucleocytoviricota* replication cycle and potential antiviral defense and resistance strategies that may be employed at various replication steps.

In addition to poorly characterized innate antiviral defenses, marine protists also possess several resistance strategies to viral infection (Fig. 2). A subset of protists, known as “grazers,” has adopted specific feeding patterns as resistance mechanisms to viral infection (54). Grazers directly ingest and inactivate marine viruses, utilizing them as a nutrient source, thereby retaining viral-derived carbon in the food web that may be distributed to higher trophic levels (54, 55). Many marine protists, such as *G. huxleyi*, have complex life cycles that feature differentiation into distinct forms. The diploid form of *G. huxleyi* is susceptible to lytic EhV infection; however, interactions with EhV trigger a shift to its non-calcified haploid form, which is resistant to infection (56, 57). Further research will be required to determine whether this kind of antiviral defense is widespread among marine protists.

Some protists resist infection through the action of integrated virophages, which can act as an inducible antiviral defense system. Virophages are small dsDNA viruses that require giant virus superinfection to replicate, which they achieve by parasitizing giant virus replication centers (58, 59). Integrated virophages can reactivate in response to host metabolic stress to inhibit giant virus replication in subsequent infection cycles (60). Metagenomic surveys have revealed that integrated virophages are abundant in protist hosts and many of these are functional, suggesting that virophages may represent a common form of antiviral defense in protists (61, 62). Collectively, these unconventional antiviral resistance strategies highlight the complex relationships between protists and their viruses.

Experimental evidence of host modulation and defense is based on a few model protists and their corresponding viruses, such as *G. huxleyi* and EhV. The small number of available infection models, while useful, is insufficient to support molecular studies of thousands of newly discovered marine viruses. Genomic approaches have been at the forefront of predicting host-virus interactions in these newly discovered viruses. However, genomics only scratches the surface of potential host-virus interactions without models to accompany them. Thus, new infection models are urgently needed to advance mechanistic understanding of virus-host interactions.

## STUDYING MIRUSVIRUS-HOST INTERACTIONS IN A MODEL ORGANISM

The discovery of mirusviruses and their chimeric properties provides tantalizing new information about the evolution of large DNA viruses (12). Shortly after the discovery of mirusviruses by metagenomics, the model heterotrophic protist *Aurantiochytrium limacinum* was shown to host two distinct mirusvirus genomes, one integrated into a sub-telomeric region on chromosomal DNA and the other located on a high copy episome (63). Both mirusvirus genomes are transcriptionally active (63) and support productive viral replication, although the episomal viral genome is far more productive than the integrated viral genome (27). Interestingly, culturing *A. limacinum* in nutrient-depleted media reduced release of viral particles, which instead accumulated between the plasma membrane and the cell wall, suggesting a regulated mechanism for virus retention until environmental conditions are favorable for release (27). While it is evident that mirusviruses infect a wide variety of hosts (64), the discovery of mirusviruses of the model protist *A. limacinum* provides outstanding opportunities to investigate virus-host interactions in the laboratory.

*A. limacinum* is a marine thraustochytrid, a group of heterotrophic protists abundant in nutrient-rich coastal regions of the oceans. Thraustochytrids play significant ecological roles in marine environments as they secrete numerous enzymes that facilitate the breakdown of organic material, influencing the cycling of carbon and other nutrients (65). Thraustochytrids also serve as vital nutrient reservoirs by synthesizing a variety of polyunsaturated fatty acids (PUFAs) that are essential for higher-order marine life (66). Thraustochytrid-derived PUFAs, such as long-chain omega-3 docosahexaenoic acid (DHA) and eicosapentaenoic acid (EPA), are also important for human health, as well as agriculture and aquaculture applications (67, 68). Many vertebrates, including humans, are unable to synthesize omega-3 fatty acids *de novo* because they lack the necessary desaturase enzymes and therefore rely on dietary sources of omega-3 fatty acids, such as fish oils (69). *A. limacinum* is considered a super-producer of PUFAs and has been successfully commercialized as a sustainable alternative for DHA production. These industrial applications for *A. limacinum* and other thraustochytrids have motivated the establishment of sophisticated methods for culture and molecular analysis, which should further enable future studies of virus-host interactions.

Metagenomics pioneered the discovery of many giant viruses, including mirusvirus, with early accounts suggesting mirusviruses are abundant and active throughout the world’s oceans (12). Mirusvirus genetic elements have since been identified in eukaryotic genomes in marine, freshwater, and even soil environments, suggesting that the host range of mirusviruses is extensive (64). However, the molecular determinants of mirusvirus infection remain unknown. Receptor binding, host modulation, evasion of antiviral defenses, and compatibility with host cell machinery are all factors that must be satisfied for the establishment of productive infections. Viruses may be flexible or stringent with these requirements, thereby influencing their lifestyle and host range. Generalist viruses, such as coronaviruses, have flexible requirements that allow for frequent host switches and spillover events, whereas stringent viruses, like herpesviruses, are host-specific and often evolve alongside their host (70–72). The identification of mirusvirus host *A. limacinum* should accelerate efforts to elucidate mirusvirus replication strategies. Comparative genomic analysis of other thraustochytrids will inform understanding of the prevalence of mirusvirus infection and could identify mirusvirus-free thraustochytrids that could serve as useful laboratory models for *de novo* infection and elucidating determinants of susceptibility.

Large DNA viruses, including mirusvirus relatives *Nucleocytoviricota* and *Herpesviridae*, reprogram host metabolism to meet the demands of viral infection. Consistent with this, initial mirusvirus genome annotations have identified several viral genes with potential functions in lipid metabolism that are ripe for functional studies (12, 38). Early indications of stress-mediated regulation of mirusvirus infection (27) suggest functional integration with host metabolism that could impact commercial production of DHA and other bioactive lipids. Industry has optimized DHA production from *Aurantiochytrium* species by altering growing conditions, carbon sources, nitrogen sources, and genetic engineering (73–76). Genetic engineering approaches often attempt to tip the balance towards DHA synthesis by weakening competing pathways and editing pathways involved in precursor synthesis, lipid efflux, and lipid synthesis, favoring DHA accumulation in the host cell (76). However, pathways for DHA biosynthesis in *Aurantiochytrium* species remain incompletely understood, while potential contributions of mirusvirus gene products remain an enticing possibility.

All viruses face the imperative of circumventing host defenses to establish infection. Protist antiviral defense systems have not been thoroughly characterized, but the *A. limacinum*–mirusvirus system may be used as a model to provide valuable insight into host defense. Detection of foreign nucleic acids may be an important component of antiviral defense in marine protists, as nucleic acid sensors such as Viperin (51) and cGAS-STING (53) have been identified in some single-cell eukaryotes. Deletion or silencing of putative antiviral defense genes is an attractive approach to elucidating their roles, as has been demonstrated by deletion of *STING* in the choanoflagellate *Monosiga brevicollis* (53). Many viruses encode immune antagonists to overcome host defenses. For example, herpesviruses encode several antagonists that block cytosolic DNA sensors, including cGAS (77). Mirusviruses may have evolved similar host subversion strategies to infect protists. Establishment of additional protist infection models will undoubtedly provide newfound opportunities to investigate the interplay between host defenses and viral immune antagonists.

The discovery of both episomal and integrated mirusvirus genetic elements in *A. limacinum* is reminiscent of herpesvirus latency in animals, where episomal viral genomes predominate, but telomere-adjacent integrated genomes have also been observed (63, 78–80). Episomal mirusvirus genomes have also been observed in other marine protists (64), indicating that this viral strategy is not unique to *A. limacinum*. Undoubtedly, additional examples of mirusvirus genomes integrated into host genomes will emerge with further study; host telomeric regions are the right place to look for these events as their recombinogenic nature likely supports both viral DNA integration and subsequent mobilization during reaction. Interestingly, integrated *Nucleocytoviricota* viral elements also exist in other eukaryotes, such as chlorophytes, amoebas, and even multicellular brown algae (81–83). Seemingly a widespread strategy, viral integrations suggest that certain endogenous viral elements are well tolerated by hosts and raise the possibility that they could be advantageous for host evolution or viral persistence. Further studies should be designed to test hypotheses about the potential symbiosis with the model system of mirusvirus and their protist hosts.

## SUMMARY AND FUTURE DIRECTIONS

**Fig 3:**
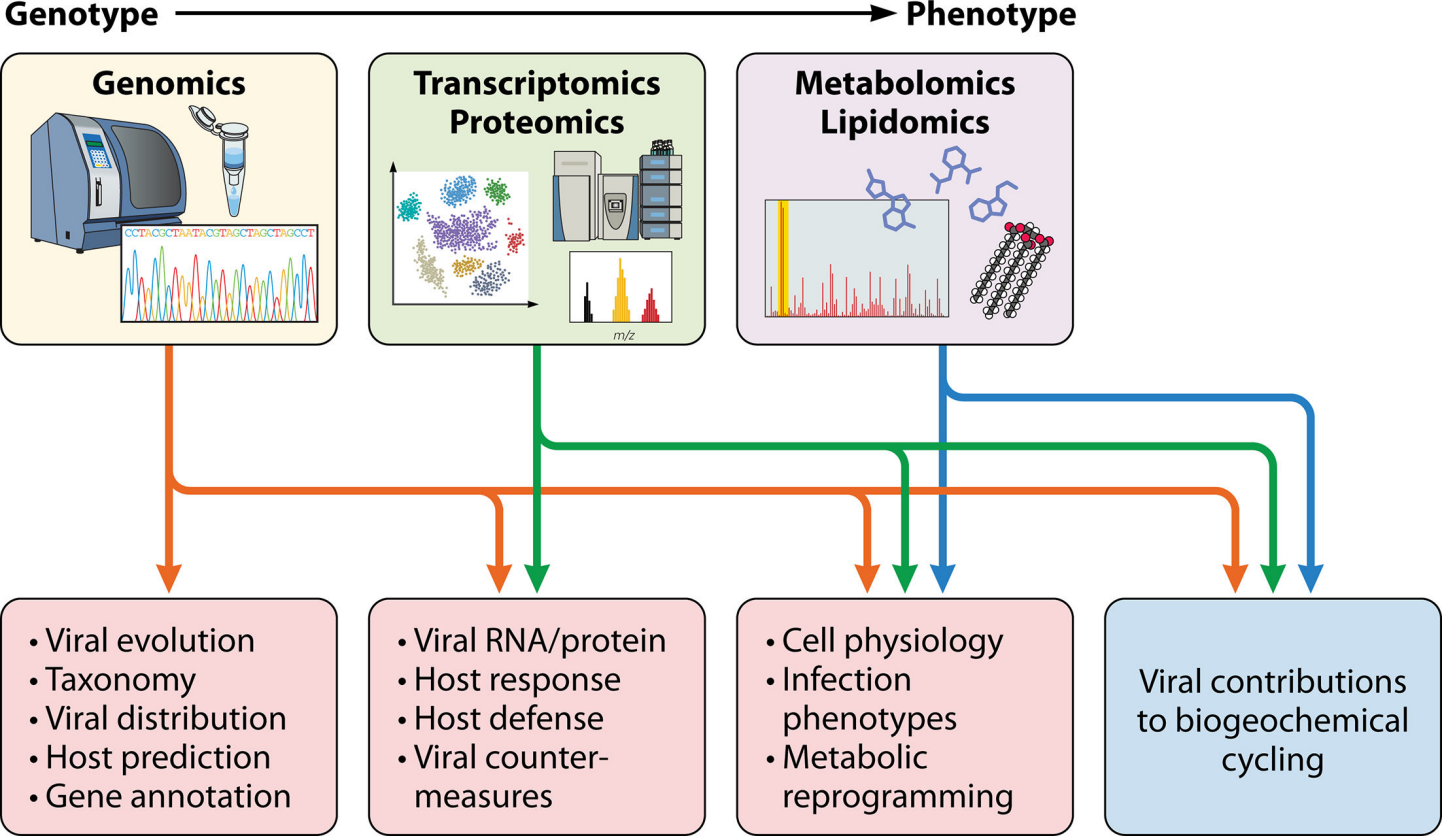
Multi-omics to characterize host-virus interactions in marine protists. The integration of genomic, transcriptomic, proteomic, metabolomic, and lipidomic data is the next step in uncovering the complexities of host-virus interactions and the impact of marine viruses on biogeochemical cycling throughout the world’s oceans.

Recent advances in comparative genomics methodology and infrastructure combined with concerted efforts to survey the world’s oceans have greatly advanced our understanding of marine viruses. These efforts have provided keen insights into taxonomic relationships and genetic interactions with hosts, including marine protists. In some cases, this rich genomic information has been complemented by laboratory infection models that provide information about molecular mechanisms of infection and regulation of virus-host interactions. Because it is not feasible to develop laboratory infection models for all marine viruses, timely elucidation of virus-host interactions requires the development and application of sensitive new methods that can be used to extend foundational knowledge. Multi-omics approaches have recently gained popularity for their ability to capture multiple informational layers in biological systems. For viral infections, multi-omics approaches can be used to understand the molecular complexities of virus-host interactions, including host antiviral defenses and viral countermeasures, which could be captured by integrating transcriptomic and proteomic information (Fig. 3). Similarly, metabolomic and lipidomic approaches can be applied to document the metabolic reprogramming of the host cells that supports resource-intensive viral replication processes. These multi-omics approaches are already useful when applied to heterogeneous populations of infected cells, but recent advances have increased sensitivity further to enable study of host cell responses at the single-cell level. While this technology is still in its infancy, one can envision that further development may one day enable single-cell -omic analysis of viral infection of marine hosts as they are collected in the field, with precise linkage to environmental and geographic data. The important role that viruses play in marine ecosystems by influencing biogeochemical cycling certainly justifies the further development of such technologies.
